# Improving stem cell-derived extracellular vesicles for better tendon and tendon-to-bone junction regeneration: strategies and future directions

**DOI:** 10.1186/s13287-026-05171-6

**Published:** 2026-07-15

**Authors:** Haining Liu, Pauline Po Yee Lui

**Affiliations:** 1 Department of Orthopaedics and Traumatology, The Chinese University of Hong Kong, Room 74037, 5/F Lui Che Woo Clinical Sciences Building, Prince of Wales Hospital, Shatin, New Territories, Hong Kong SAR China; 2 InnoHK Center for Neuromusculoskeletal Restorative Medicine, Hong Kong Science Park, Shatin, New Territories, Hong Kong SAR China

**Keywords:** Exosomes, Extracellular vesicles, Genetic modification, Preconditioning, Tendinopathy, Tendon healing, Three-dimensional culture

## Abstract

Chronic tendinopathy is a debilitating tendon overuse disorder characterized by localized tenderness, swelling, and pain, significantly impairing physical function. It is particularly common among the overuse and aging populations. Traditional treatment options, including conservative therapies and surgical interventions, often yield limited success. Recent studies indicate that extracellular vesicles (EV) derived from stem cells provide a promising therapeutic avenue for healing of tendon and tendon-to-bone junction (TBJ) injuries associated with chronic tendinopathy. Their unique properties, such as higher cargo stability and the ability to serve as carriers for targeted drug delivery, position them as ideal candidates for tendinopathy treatment. However, the low yield of EVs presents challenges for clinical applications. This review systematically review various strategies to enhance EV yield and function, including preconditioning stem cells through biophysical, biological, or chemical means; genetic engineering of stem cells; in vitro loading of proteins or drugs and surface modification of EVs; and modifying the stem cell culture environment, particularly through three-dimensional (3D) culture techniques. Emphasis is placed on scaffold-free methods, scaffold-based methods, 3D printing, spinner flasks, and bioreactors, which can potentially improve EV yield and functions for tendon and TBJ regeneration. The review also summarizes relevant preclinical data and explores the molecular mechanisms underlying enhanced EV yield and functions, as well as the mechanisms of EVs on tendon and TBJ repair. Future research directions include investigating various EV enhancement strategies in models of degenerative tendon injury, studying the underlying molecular mechanisms, establishing cost-effective methods for scalable EV production, optimizing EV treatment protocols for clinical translation, and exploring combination strategies to enhance therapeutic EV production and function.

**Graphical Abstract:**

**Figure:**
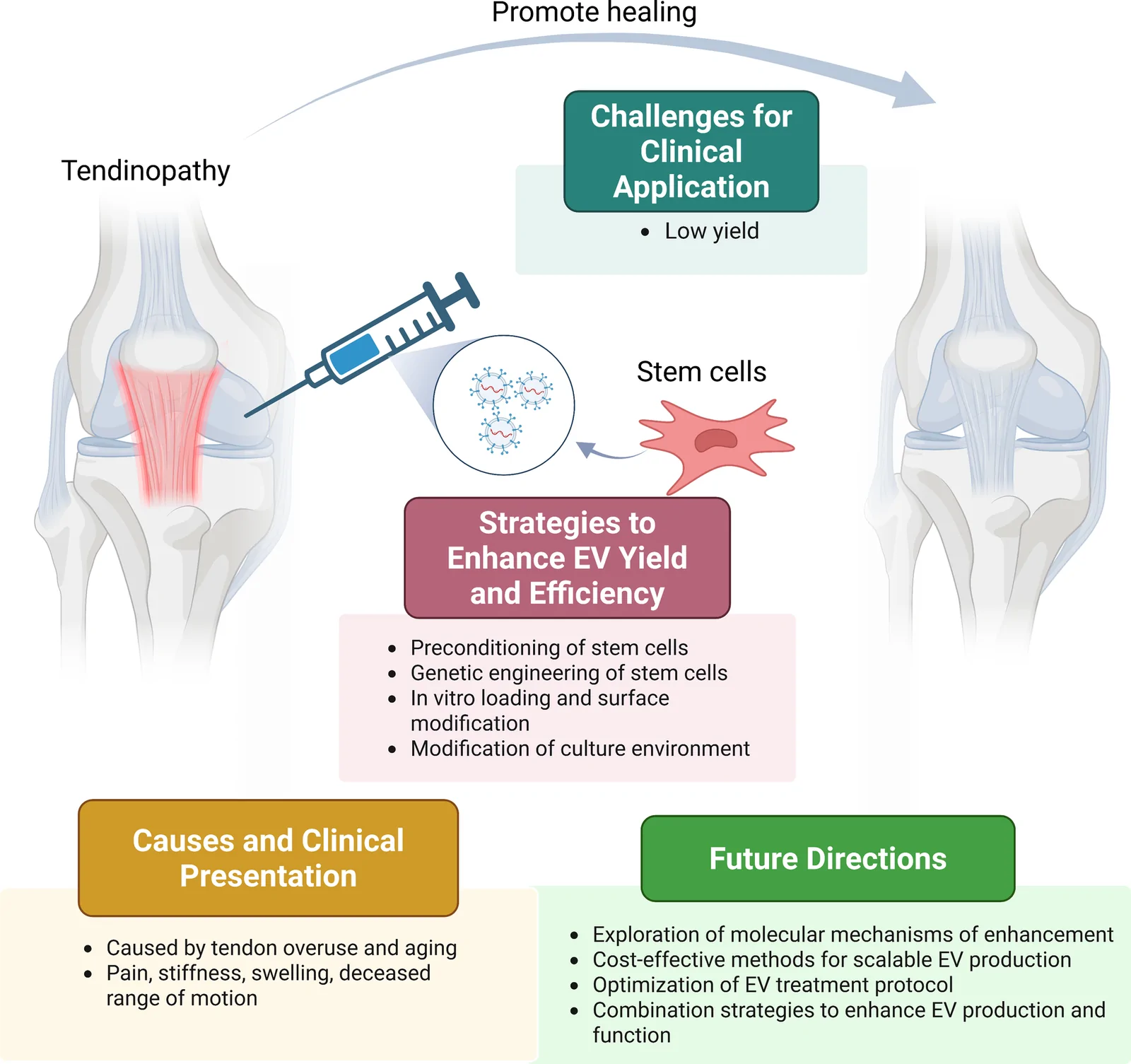
Created in BioRender. https://BioRender.com/198vi3b

## Chronic tendinopathy and current treatment limitations

### Overview of tendinopathy

Chronic tendinopathy is a debilitating tendon overuse disorder mainly caused by tendon overuse and aging. It is characterized by local tenderness, swelling, and pain in the affected tendons, which limits physical function. Over time, the tendon degenerates and may rupture in certain types of tendinopathy. Common sites include the rotator cuff, extensor carpi radialis brevis of the elbow, patellar tendon, and Achilles tendon.

Chronic tendinopathy is common, with worldwide incidence of tendon rupture ranging from 80 to 90 cases per 100,000 inhabitants, equivalent to 6 to 7 million annually, as reported in a recent systematic review and meta-analysis [1]. Specifically, Achilles tendinopathy affects about 6% of the general population and up to 50% of elite endurance runners over their lifetimes [2]. Rotator cuff tendinopathy affects 30% of the general population [3]. About 30% of the elderly age over 60 have a rotator cuff tear and this increases to 62% in those over 80 [4].

### Current treatments and stem cell-based therapies

Tendinopathy can occur in various regions of the tendon, including the mid-substance and the tendon-to-bone junction (TBJ). While conservative treatments for mid-substance and TBJ tendinopathy may be similar, the prognosis for TBJ is more variable due to the unique anatomical, biomechanical, and physiological characteristics of the enthesis. The tendon is a dense, collagen-rich connective tissue that transmits forces from muscle to bone. In contrast, the enthesis comprises four structurally graded transition zones (tendon, non-mineralized proteoglycan-rich fibrocartilage, mineralized fibrocartilage, and bone). This complex structure is crucial for force transmission and preventing stress concentration. Consequently, the cellular composition, extracellular matrix, and mechanical function of the TBJ differ significantly from those of the tendon mid-substance [5]. Fibrous scar tissue forms, and the fibrocartilage zone at the enthesis often fails to regenerate after tendon mid-substance and insertion repair, respectively, increasing the risk of re-rupture. Therefore, treatment goals for mid-substance and TBJ tears differ, necessitating tailored approaches for effective healing.

Both conservative therapies and surgical interventions have shown limited effectiveness. Exercise is the first-line conservative treatment; however, its effectiveness varies among patients and is often hindered by adherence issues [6]. Classical anti-inflammatory drugs, such as non-steroidal anti-inflammatory drugs (NSAIDs) and steroids, provide only short-term pain relief [7]. Despite their immunosuppressive benefits, these medications can adversely affect tendon cell survival, metabolism, senescence, differentiation, and healing [8–14].

Current treatments are unsatisfactory due to a limited understanding of the underlying mechanisms. Tendon overuse triggers an inflammatory response [15], leading to erroneous differentiation of tendon-derived stem/progenitor cells (TDSCs) [16–18]. By targeting tenogenesis alongside excessive inflammation, we can develop more effective solutions for this chronic condition.

There is a growing interest in using stem cells to treat tendinopathy due to their immunomodulatory and tissue regenerative properties. Preclinical studies have demonstrated positive effects of stem cells for this condition [19–20]. However, prospective controlled studies yielded mixed results regarding the use of mesenchymal stromal cell (MSC)-based therapies for this condition [21–22]. Additionally, ethical considerations, safety, and quality control of stem cells remain critical issues that need to be addressed for these therapies.

This review aims to systematically review the roles and molecular mechanisms of extracellular vesicles (EVs) in promoting tendon and TBJ repair, as well as various strategies to enhance EV yield and function. Emphasis is placed on various three-dimensional (3D) culture techniques and their mechanisms, which can potentially improve EV yield and functions. Limitations of current studies and future research directions are also discussed. This review involves a systematic search of titles and abstracts in PubMed using the keywords (“Tendon”) AND (“extracellular vesicles” OR “exosomes”) from 25 Apr to 28 May 2025. The keyword “Tendon” was selected to encompass papers related to both “tendon” and “tendon-to-bone junction”, as the tendon is integral to the structure of the tendon-to-bone junction. We reviewed each paper to identify relevant studies and included relevant literature on various EV enhancement strategies from other research areas to highlight research gaps in tendon and TBJ field.

## Role of extracellular vesicles in tendon and tendon-to-bone junction healing

### Definitions and properties of extracellular vesicles

EVs are lipid bilayer particles secreted by various cell types that play a crucial role in intercellular communication. They carry diverse cargo of proteins, lipids, and nucleic acids that facilitate the transfer of biological information between cells. Based on sizes and biogenesis, EVs are classified into three categories: exosomes (50–150 nm, formed by the fusion of the cell membrane and multivesicular bodies), microvesicles (100–1000 nm, produced by outward budding of the cell membrane), and apoptotic bodies (1–5 μm, generated by outward blebbing of the cell membrane). Most current tissue engineering research focuses on exosomes. For consistency, we will refer to EVs throughout this review.

EV-derived from stem cells offer several advantages over their parent cells in clinical translation [23]. As non-cellular entities, they are less immunogenic, mitigating ethical concerns and reducing risks associated with stem cell therapies. Their ease of isolation and storage further enhances their convenience for clinical applications. Additionally, the lipid bilayer of EVs improves cargo stability while minimizing toxicity during delivery. Furthermore, EVs can effectively transport therapeutic molecules with low oral bioavailability and poor epithelial penetration to target cells.

### Extracellular vesicles for tendon and tendon-to-bone junction repair

There is an interest in using stem cell-derived EVs as a cell-free alternative for treating tendinopathy. For example, intratendinous injection or transplantation of EVs derived from TDSCs (TDSC-EVs) has been shown to promote tendon repair in a collagenase-induced rat model of Achilles tendinopathy [24–25]. Furthermore, injecting EVs isolated from human induced pluripotent stem cell-derived mesenchymal stromal cells (iPSC-MSCs) alleviates pain and enhances tendon healing in a rat model of carrageenan-induced quadriceps tendinopathy [26]. Moreover, treating injured supraspinatus tendons with EVs from adipose-derived stromal cells (ADSC-EVs) regulates macrophage polarization and promotes tendon healing compared to a saline control in an overuse tendinopathy mouse model [27]. Additionally, transplantation of EVs from human umbilical cord MSCs (HUMSC-EVs) reduces inflammation and tendon adhesion in a rat Achilles tendon injury model [28]. A recent study highlights that clinical-grade HUMSC-EVs suppress T-cell proliferation and promote tendon and TBJ repair in a large ovine infraspinatus tendon and enthesis injury model [29]. For a comprehensive overview of the effects of EVs in treating tendon and TBJ injuries, including tendinopathy, please refer to the published review articles [19, 23, 30].

### Molecular mechanisms in tendon and tendon-to-bone junction regeneration

MSC-derived EVs (MSC-EVs) enhance cell proliferation, migration, and angiogenesis, and suppress oxidative stress, apoptosis, and extracellular matrix (ECM) degradation, while modulating stem cell differentiation and the inflammatory responses of macrophages [23–25, 27, 31–43]. Additionally, MSC-EVs increase collagen deposition, decrease fatty infiltration, and promote angiogenesis during healing [33, 38–40, 44–45]. Although the precise active components responsible for these effects remain unclear, MSC-EVs are rich in miRNA and proteins critical for tendon and TBJ healing.

miRNA sequencing reveals elevated levels of miR-29a-3p in HUMSC-EVs compared to parent cells, which downregulate phosphatase and tensin homolog (PTEN), activate protein kinase B (Akt) phosphorylation, leading to upregulation of mammalian Target of Rapamycin (mTOR) signaling and transforming growth factor-beta 1 (TGF-β1) synthesis in target TDSCs [45]. Additionally, HUMSC-EVs express low levels of miR-21a-3p, which reduce the expression of p-p65, cyclooxygenase-2 (Cox-2), collagen 3 A 1 (Col3A1), and alpha-smooth muscle actin (α-SMA) in fibroblasts, thereby decreasing inflammation and fibrosis in rat tendons [27]. Another study has shown that HUMSC-EVs promote tendon healing via miR-27b-3p-induced inhibition of ARHGAP5, which activates Ras homolog family member A (RhoA) and enhances the proliferation and migration of injured tenocytes [46]. Additionally, TDSC-EVs contain miR-144-3p, which enhances tendon repair by regulating tenocyte proliferation and migration [38]. Furthermore, TDSC-EVs promote Achilles tendon repair via upregulation of miR-145-3p, enhancing proliferation, migration, and tenogenic marker expression while decreasing osteogenic markers in TDSCs [47]. In another study, EVs derived from scleraxis (Scx)-overexpressing platelet-derived growth factor receptor alpha-positive (PDGFRα(+)) BMSCs reduce osteoclastogenesis and improve the mechanical strength of the TBJ via exosomal miR-6924-5p delivery in a rat extra-articular tendon-bone tunnel model [48]. The therapeutic effects of small and large EVs on tissue repair can vary due to their different contents. For instance, the administration of ADSC exosomes demonstrates superior effects compared to microvesicles in promoting healing in a rat model of collagenase-induced Achilles tendinopathy [49]. miRNA sequencing reveals that the expression levels of miR-29a, miR-21-5p, and miR-148a-3p, known to promote tendon healing, are significantly enriched in ADSC-derived exosomes compared to microvesicles [49].

**Fig. 1:**
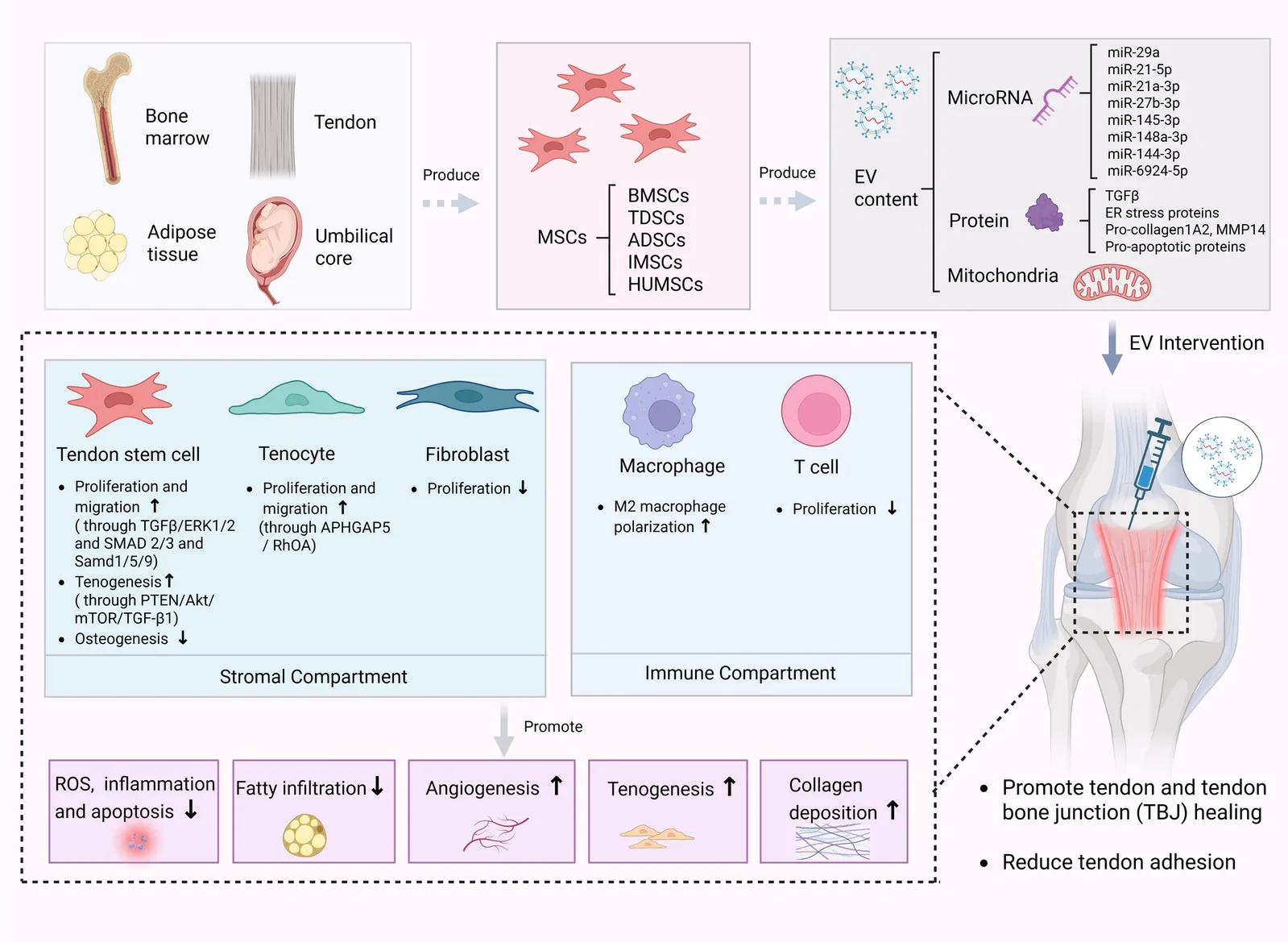
Mechanisms of action of extracellular vesicles in tendon and tendon-to-bone junction healing. Created in BioRender. https://BioRender.com/vvygpsq

EVs are also rich in proteins that regulate proliferation, migration, oxidative stress, apoptosis, and ECM remodeling. Notably, TDSC-EVs carry higher levels of TGF-β compared to their parent cells [50]. The delivery of exosomal TGF-β activates the extracellular signal regulated kinase ½ (ERK1/2) signaling pathway and SMAD Family Member 2/3 (Smad2/3)-mediated matrix metalloproteinase 2 (MMP2) expression, which may explain the increased proliferation and migration of target TDSCs [50]. Similarly, Liu et al. reported that ADSC-EVs promoted TDSC proliferation, migration, and tenogenic differentiation by activating the Smad2/3 and Smad 1/5/9 signaling pathways in vitro, as well as enhancing tendon healing in vivo, suggesting the involvement of the TGF-β signaling pathway [50]. HUMSC-EVs contain endoplasmic reticulum stress proteins, such as glucose-regulated protein 7 (GRP7) and C/EBP homologous protein (CHOP), as well as pro-apoptotic proteins like Bcl-2 associated X protein (Bax), which may contribute to their anti-adhesive effects in traumatic tendon injuries [51]. Additionally, EVs derived from bone marrow-derived stromal cells (BMSC-EVs) express pro-collagen1A2 and matrix metalloproteinase 14 (MMP14), both crucial for tendon ECM remodeling. The presence of pro-collagen 1A2 on the EV surface is essential for its functions, as pretreatment with trypsin nullifies its effects on tendon cell proliferation, migration, and collagen type I expression. This suggests that the biological activities of these EVs depend on the interaction of their membrane-bound proteins with recipient tendon cells [33]. In addition, functional mitochondrial transfer to replace damaged mitochondria in target cells has recently been identified as a new mechanism mediating the immunomodulatory effects of EVs. In one study, inhibiting mitochondrial function in EVs—achieved by treating the donor BMSCs with a high concentration of rhodamine 6G—nullified their ability to transfer functional mitochondria to macrophages and supraspinatus muscles, promote M2 macrophage polarization, and exert anti-inflammatory and antioxidant effects [43]. This impairment also reduced their capacity to mitigate muscle degeneration and scarring following rotator cuff tendon and nerve injuries [43]. A summary of the mechanisms of EVs in tendon and TBJ healing, based on existing literature, is shown in Fig. 1.

## Strategies to enhance extracellular vesicle yield and function

**Fig. 2:**
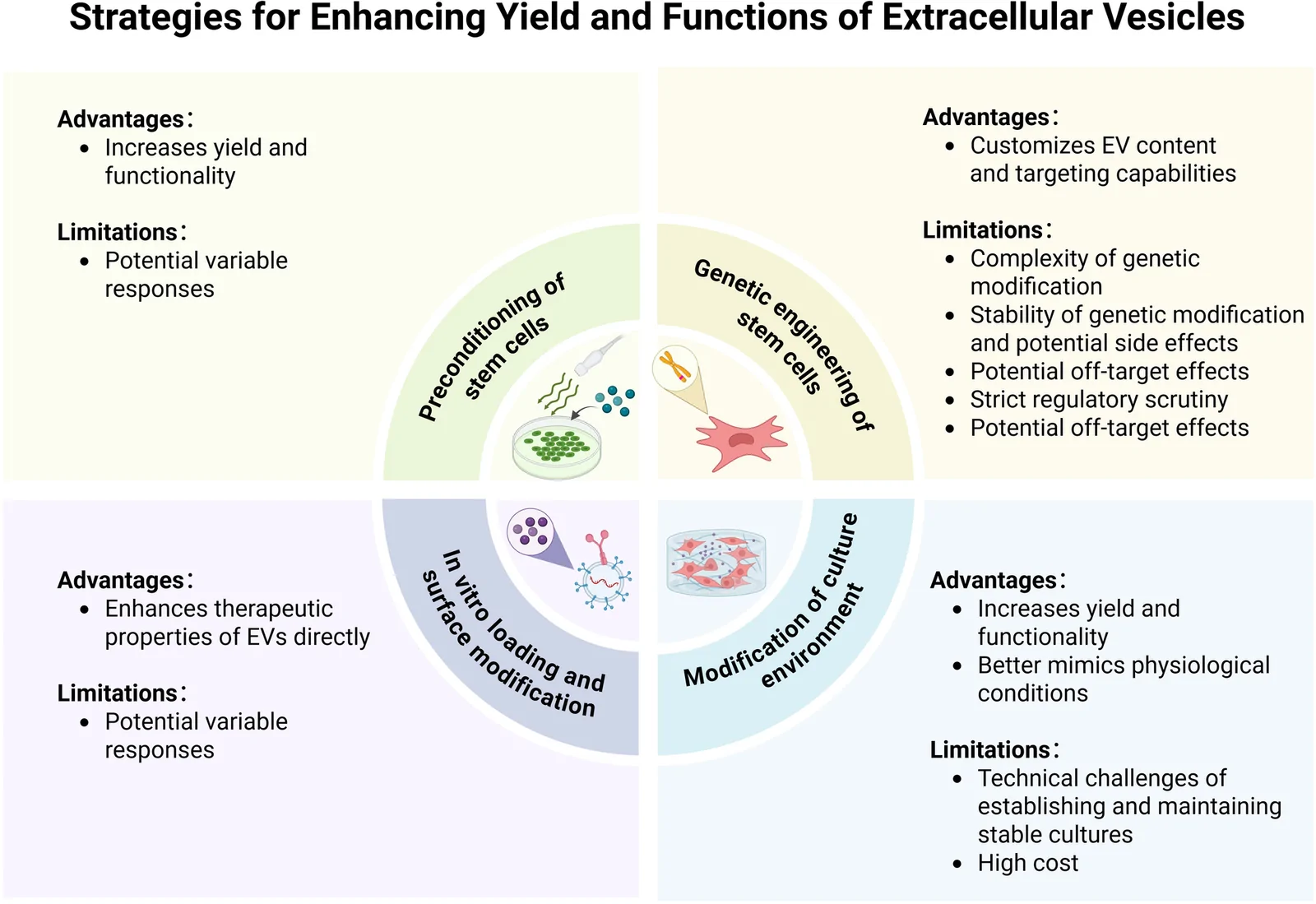
Strategies for enhancing yield and functions of extracellular vesicles. Created in BioRender. https://BioRender.com/5hu1trm

While EVs offer significant benefits for tendon healing, their low yield limits their effectiveness in tendon and TBJ repair. To enhance both yield and biological functions, various strategies are employed. These strategies not only improve treatment efficacy but also facilitate clinical translation. They can be broadly categorized into 4 types: preconditioning of stem cells, genetic engineering of stem cells, in vitro loading and surface modification, and modification of culture environment. Each method has its advantages and limitations, which, along with relevant published findings on tendon and TBJ repair, are discussed in the following sections and summarized in Fig. 2; Table 1.

### Preconditioning of stem cells

Priming stem cells with biophysical, biological, and chemical methods enhances the yield and therapeutic potential of EVs to support tendon and TBJ regeneration.

#### Biophysical priming

Stem cells respond remarkably to biophysical stimuli, which activate mechanosensitive and voltage-gated ion channels, as well as the integrin-actin-cytoskeletal network. This triggers calcium ions influx and cytoskeletal reorganization, activating key signaling pathways, such as MAPK and PI3K/Akt. These pathways, in turn, stimulate the production of growth factors and cytokines that support cell survival, migration, and stem cell differentiation [52].

A notable example is using low-intensity pulsed ultrasound (LIPUS) to precondition BMSCs. EVs derived from LIPUS-pretreated BMSCs (LIPUS-BMSC-EVs) demonstrate enhanced fibrocartilage regeneration and reduced fatty infiltration in a mouse rotator cuff repair model compared to EVs isolated from untreated BMSCs (BMSC-EVs) [53]. This improvement stems from an enrichment of chondrogenic miRNAs, particularly miR-140, and reduced adipogenic miRNAs.

Similarly, EVs from magnetically actuated BMSCs outperform those from untreated BMSCs in promoting TBJ healing during anterior cruciate ligament reconstruction [54]. In vitro studies show that these magnetically enhanced EVs have higher levels of specific miRNAs, including miR-21-5p, miR-25-3p, miR-92a-3p, miR-122-5p, miR-125b-5p, and miR-150-5p. These EVs are more effectively internalized by recipient cells and significantly promote fibroblast proliferation, migration, and fibrogenesis. The effects are mediated partly by exosomal miR-21-5p, which downregulates SMAD7, a key regulator of fibrosis and tissue repair.

In another study, pre-treating ADSCs with a static magnetic field enhances their proliferation and colony-forming potential. This approach also increases the yield of microvesicles enriched with growth factors such as vascular endothelial growth factor (VEGF), bone morphogenetic protein-2 (BMP-2), and p53, while reducing pro-inflammatory tumor necrosis factor-alpha (TNF-α) levels compared to untreated cells [55]. These pre-treated microvesicles show significant potential for tissue regeneration, emphasizing the value of biophysical preconditioning in promoting healing of musculoskeletal disorders and tendon injuries.

#### Biological priming

Biological priming involves using growth factors and cytokines to amplify the antioxidant, anti-inflammatory, and regenerative capacities of produced EVs.

To effectively utilize biological priming to enhance EV production and function, it is essential to understand how stem cells respond to biological signals, particularly inflammation, which occurs after tendon injuries. An inflammatory environment is crucial for MSCs to initiate reparative and immunosuppressive responses. Notably, MSCs can polarize into two distinct phenotypes—MSC1 and MSC2—based on Toll-like receptor (TLR) stimulation [56]. TLR4 stimulation in the absence of an inflammatory environment yields the pro-inflammatory MSC1 phenotype, while TLR3 stimulation in the presence of inflammation produces the anti-inflammatory MSC2 phenotype [56]. This polarization underscores the need to control the priming conditions carefully.

Demonstrating this principle, priming MSCs with inflammatory cytokines, such as interferon-gamma (IFN-γ) or TNF-α, amplifies their secretion of immunomodulatory and regenerative growth factors. This approach increases the production of soluble factors like cyclooxygenase-2 (Cox-2), human growth factor (HGF), TGF-β, and indoleamine 2,3-dioxygenase (IDO), all of which promote M2 macrophage polarization [57–59]. Similarly, Dong et al. showed that electrospun polycaprolactone/silk fibroin (PCL/SF) scaffolds functionalized with ECM derived from IFN-γ-primed BMSCs (iECM) effectively stimulated the polarization of M1 to M2 macrophages in vitro, reducing the expression of inflammatory cytokines [60]. In vivo, these iECM-functionalized scaffolds effectively reduced tendon adhesion in a rat model of Achilles tendon injury, outperforming scaffolds modified with ECM from untreated BMSCs [60].

The benefits of priming MSCs extend to the EVs they produced. Preconditioning BMSCs with IFN-γ and TNF-α generates EVs with enhanced anti-inflammatory capabilities, mediated by elevated Cox-2 expression in the EVs [61]. Similarly, preconditioning HUMSCs with interleukin-1beta (IL-1β) enhances EV-mediated transfer of miR-146a to macrophages, promoting M2 polarization [62]. In the context of tendon healing, EVs derived from IFN-γ-primed ADSCs effectively inhibit inflammatory activity in macrophages and enhance tendon cell proliferation and collagen production, leading to improved outcomes compared to EVs from naïve ADSCs [32, 63]. This enhanced effect is mediated by increased expression of miR-147b in EVs from IFN-γ-primed ADSCs, which modulates the inflammatory responses in macrophages by inhibiting the Toll-Like Receptor 4/Nuclear Factor kappa-light-chain-enhancer of activated B cells (TLR4/NF-kB) signaling pathway [63].

In addition to inflammatory cytokines, priming strategies can utilize other biological factors. For example, melatonin pretreatment modulates the circadian rhythm of ADSCs, increasing yield and enhancing anti-inflammatory capacity of EVs [64]. These circadian rhythm-regulated EVs inhibit M1 macrophage polarization through platelet factor 4 transfer, which enhances the cyclic adenosine monophosphate (cAMP) signaling pathway. The melatonin-primed EVs also improve TBJ regeneration in a rotator cuff repair model when delivered locally using a microneedle system [64]. Furthermore, EVs derived from kartogenin, a chondrogenic factor, preconditioned BMSCs promote fibrocartilage regeneration in a rat model of chronic rotator cuff repair [65].

Given the impact of oxidative stress and inflammation on tendon injuries, strategies aimed at mitigating these factors while enhancing TDSC function are particularly promising [66]. For instance, EVs isolated from eugenol (an antioxidant)-primed BMSCs protected TDSCs against oxidative stress. The transplantation of eugenol-primed EV-treated TDSCs significantly promoted tenogenesis and matrix regeneration during tendon healing compared to the administration of TDSCs treated with unprimed EVs [67]. Similarly, EVs derived from hydroxycamptothecin-primed HUMSCs, enriched with endoplasmic reticulum stress and apoptosis-related proteins (GRP78, CHOP, Bax), enhanced effectiveness in inhibiting myofibroblast differentiation in fibroblasts [51]. Transplantation of the primed EVs to the injury site improved the histological score, although there was no improvement in the adhesion score or mechanical properties of the injured tissue compared to unprimed EVs in an Achilles tendon injury model [51].

#### Chemical priming

Chemical priming leverages the ability of chemical compounds to influence cellular processes, thereby enhancing the properties of secreted EVs.

One example of this approach involves using lipopolysaccharide (LPS) to precondition MSCs. Studies show that EVs derived from LPS-preconditioned MSCs exhibit superior abilities to upregulate anti-inflammatory cytokines and facilitate M2 macrophage polarization compared to EVs derived from untreated MSCs [68]. This enhanced immunomodulatory effect is mediated by the unique expression of let-7b in EVs derived from LPS-preconditioned MSCs [68]. Injecting LPS-primed EVs into the wound edge alleviates inflammation and promotes wound healing compared to the injection of unprimed EVs [68]. However, the effects of LPS-primed EVs on tendon and TBJ repair remain unexamined and warrant further research.

Another promising chemical agent is bioactive glass, a material composed of SiO_2_, Na_2_O and CaO, and P_2_O_5_. Its ionic products promote proliferation and differentiation of various cell types, modulate macrophage polarization, enhance cell paracrine functions, and promote vascularization [69–70]. Xu H et al. utilized bioactive glass to prime ADSCs, enhancing the anti-inflammatory and angiogenic effects of their EVs [71]. EVs derived from these preconditioned ADSCs exhibited a distinct miRNA profile with enhanced anti-inflammatory and regenerative effects. Specifically, these modified EVs promoted macrophage polarization, macrophage-mediated angiogenesis, and Achilles tendon repair in vitro and in vivo. These benefits are likely mediated by the upregulation of miR-199b-3p and miR-125a-5p [71], further illustrating the targeted manipulation of EV function through chemical priming.

*Challenges and considerations* While preconditioning stem cells with biophysical, biological, and chemical stimuli improves EV yield and therapeutic effects, responses can be variable, requiring optimization of preconditioning parameters.

In summary, preconditioning stem cells with biophysical, biological, and chemical stimuli enhances the quality and quantity of EVs for tendon and TBJ repair. Further studies are needed to verify the consistency of these responses and to fully elucidate the detailed mechanisms underlying these effects. This is crucial for translating these promising approaches into reliable and effective clinical therapies.

### Genetic engineering of stem cells

Genetic engineering has emerged as a powerful tool to enhance both the therapeutic functions and targeted delivery of stem cell-derived EVs. By manipulating the genetic makeup of the parent cells, researchers can precisely control the composition and properties of the EVs produced, improving regenerative outcomes.

One approach involves modifying the exosomal payload. For example, An et al. demonstrated that EVs derived from miR-337-3p overexpressing TDSCs downregulated caspase 3 and protected tenocytes from apoptosis [72]. Similarly, Yao et al. showed that overexpressing miR-29a-3p in HUMSCs resulted in EVs with elevated levels of this miRNA, promoting tendon healing through PTEN downregulation, Akt activation, and subsequent increased mTOR signaling and TGF-β1 production in target TDSCs [45]. Conversely, reducing specific miRNAs can also be beneficial. Yao et al. found that transfecting HUMSCs with a miR-21a-3p antagonist led to EVs with further reduced levels of miR-21a-3p [27], significantly improving anti-inflammatory and anti-fibrotic effects by further reducing the expression of p-p65, Cox-2, Col3A1, and α-SMA in TGF-β1-treated fibroblasts. These changes may further reduce tendon adhesion and promote healing in tendon injury models, as evidenced by the decreased miR-21a-3p levels in tendon tissues after treatment with naive EVs. Furthermore, Feng et al. demonstrated that EVs derived from PDGFRα(+) BMSCs engineered to overexpress *Scx* enhanced TBJ healing by delivering miR-6924-5p, which reduced osteoclastogenesis and strengthened tendon-to-bone attachment [48].

Beyond modifying the EV payload, genetic engineering can direct EVs to specific tissues or cell types, enabling targeted delivery. This involves engineering source cells to express specific proteins and peptides on the EV surface. As EVs inherit their lipid bilayer from the membranes of their originating cells, altering the parent cells offers a strategy to modify EV surface characteristics. This can be accomplished by constructing fusion gene vectors that encode EV-associated membrane proteins, such as modified negative regulatory factor (Nef) [73], CD9 [74–75], CD63 [76–77], or lysosomal associated membrane protein 2B (LAMP2B) [78–80]. Cui et al. exemplified this approach by functionalizing the surface of TDSC-derived EVs with a collagen-binding domain (CBD) through CD63-CBD overexpression in the parent TDSCs [81]. These modified TDSC-EVs were then tethered to decellularized bovine tendon sheets for sustained release. The resulting construct significantly enhanced proliferation and tenogenic differentiation of TDSCs and BMSCs in vitro [81]. In vivo, transplanting the construct into an Achilles tendon injury model promoted endogenous stem cell recruitment, M2 macrophage polarization, collagen deposition, and tenogenesis, while reducing inflammatory cytokine expression, and improving biomechanics and the Achilles tendon functional index [81]. Another study overexpressed CBD-LAMB2B in CD26^+^ TDSCs, a distinct population of peritendon stem progenitor cells involved in tendon repair and heterotopic ossification [82]. This was accomplished using a lentivirus system, with the cells pretreated with the BMP type I receptor inhibitor LDN193189 before EV isolation [83]. The researchers reported that CBD facilitated the sustained release of modified EVs from the scaffold. The 3D-bioprinted scaffolds containing LDN193189-pretreated CBD-modified EVs demonstrated superior effects in promoting segmental Achilles tendon repair and preventing heterotopic ossification in aged rats compared to scaffolds loaded only with LDN193189-pretreated EVs, which were more effective than the untreated EVs in scaffold group and the injury-only group [83].

*Challenges and considerations* Genetic modification of stem cells to enhance EV production and functions introduces complexities and potential risks. Depending on the regulatory mechanisms, the target proteins overexpressed in parental cells may not effectively incorporate into EVs. Besides, the process requires advanced techniques such as CRISPR/Cas9 or viral vectors, each demanding precise protocols and optimization, which can be challenging to achieve consistently. Furthermore, different cell types may require specific vectors or methods for successful transfection. Ensuring stability of genetic modification over time is also essential, as off-target effects and unintended mutations may increase tumorigenic and immunogenic risks. Additionally, genetic modification of stem cells is subject to strict regulatory scrutiny, potentially delaying research and clinical applications.

In summary, while genetic engineering offers exciting possibilities for enhancing EV-based therapies, careful consideration must be paid to technical challenges, regulatory hurdles, and potential risks associated with this approach. Further research is needed to optimize these strategies and ensure their safe and effective clinical translation.

### In vitro loading and surface modification

In vitro loading and surface modification involve directly manipulating EVs post-isolation, either by introducing specific proteins, miRNAs, or drugs into their interior or by altering their surface to improve targeting, uptake, and circulation. These strategies significantly enhance EV efficacy in tendon and TBJ healing.

*In vitro loading techniques* Methods for loading target proteins and drugs into EVs range from simple incubation to advanced techniques. These include chemical methods utilizing lipophilic agents, electroporation, sonication, freeze-thaw cycles, gradient pH regulation, and microfluidics.

For example, Wang et al. bypassed the pre-treatment of MSCs with kartogenin [65] and loaded kartogenin directly into BMSC-EVs using sonication [84]. This approach enhanced chondrogenesis of target BMSCs via upregulation of mosaic protein domain (*Mospd*) and augmented fibrocartilage regeneration compared to the transplantation of unmodified EVs in a rat rotator cuff injury model, demonstrating the efficacy of direct drug loading.

Simple incubation has low loading efficiencies and it is difficult to control the amount of drug loaded into the EVs. On the other hand, the loading efficiencies of the active cargo loading methods depends on the hydrophilicity and molecular weight of the drug being loaded. The EV size may increase after drug loading, affecting the integrity of EV membranes.

*Surface modification* Modifying EV surface enhances targeting, uptake, and circulation half-life. This can be achieved by attaching specific ligands, such as antibodies and peptides, to the EV surface. Furthermore, modifying the lipid composition of EVs can influence their interaction with target cells, enhancing uptake of the loaded agents. Coating EVs with specific polymers, such as polyethylene glycol (PEG) or peptides can further increase half-life and reduce immunogenicity, thereby improving drug delivery.

*Hybrid nanocarriers* There is growing interest in modifying MSC-EVs to combine the advantages of native EVs and synthetic nanoparticles to improve cargo loading efficiency. Pareja Tello et al. developed hybrid ADSC-EVs and lipid nanoparticles using a microfluidic-sonication technique to enhance the delivery of collagen type I mRNA into pathological TDSCs [85]. The sonic wave created compressive stress on the lipid membrane and ruptured the EV membrane, leading to the reassembly of the lipid structures with the EV material. These hybrid nanocarriers retained EV surface protein markers and encapsulated mRNA. The production of procollagen type I carboxy-terminal peptide (PICP) in TDSCs after hybrid nanocarrier treatment increased by 2-fold compared to the untreated cells, and was similar to lipid nanoparticles loaded with collagen type I mRNA, highlighting the potential of these hybrid systems [85].

*Challenges and considerations* Molecular size, charge, and hydrophobicity significantly impact protein or drug incorporation into EVs, affecting loading efficiencies. Furthermore, variations in loading efficiency between batches complicate research and clinical applications. The release kinetics can also vary based on the nature of the proteins and drugs, as well as EV lipid composition, potentially complicating treatment outcomes.

In summary, in vitro loading and surface modification techniques offer promising avenues for tailoring EVs for specific therapeutic applications. However, careful optimization and characterization are essential to address technical challenges and ensure consistent quality in EV-based therapies.

### Modification of culture environment

The composition and therapeutic efficacy of EVs are significantly influenced by the culture conditions of their parent cells. Therefore, modifying the culture environment represents a useful strategy for optimizing EV production and quality. Key environmental factors that impact the yield and functions of EVs include oxygen levels, and the adoption of 3D culture systems.

#### Oxygen levels

Culturing MSCs under hypoxic conditions (1–5%) enhances expansion, stemness, and regenerative capacity, ultimately improving tendon repair [86–88]. Mass spectrometry analysis revealed that EVs derived from hypoxic ADSCs have higher levels of MMP2, collagen type 6 A (COL6A), cathepsin D (CTSD), and tenascin C (TNC), which are critical for ECM homeostasis [89]. Furthermore, EVs derived from hypoxia-stimulated BMSCs promote angiogenesis and augment tendon graft-to-bone tunnel healing in anterior cruciate ligament reconstruction compared to EVs isolated from BMSCs cultured under normoxia [41]. Further research is needed to investigate the effects of MSC-EVs produced under hypoxia on tendon and TBJ healing.

#### 3D culture methods

To better replicate the complex 3D tissue environment, researchers are adopting 3D culture methods to more accurately mimic the native tissue environment. Traditional two-dimensional (2D) cultures fail to mimic crucial cell-cell and cell-matrix interactions [90–91], adversely affecting functions of stem cells and their secretome such as stemness, survival, proliferation, cellular senescence, multipotency, angiogenesis, and immunomodulation [92–98]. This limitation hampers therapeutic performance and large-scale EV production needed for clinical applications. Culturing stem cells in 3D to form organoids or spheroids is gaining interest for increasing EV yield and efficacy [96, 99].

*Spheroids vs. organoids* Spheroids aggregate single cells into multicellular structures, while organoids differentiate stem cells into complex, multicellular tissues in vitro. Spheroids create a physiologically relevant environment through gradients of nutrients and factors, enhancing cell survival, stemness, and multipotency. They also alter EV biology, resulting in more pro-angiogenic and anti-inflammatory factors compared to stem cell monolayers [100–104]. Despite demonstrated capacity to improve healing in various tissues, such as bone and cartilage [105–106], the application of 3D spheroids in tendon repair remains relatively unexplored.

Organoid culture involves exposing stem cells to specific growth factors and signaling molecules in a multi-step process that directs them to differentiate into the desired cell types. The differentiated cells are then induced to aggregate into a 3D structure using various methods shared for spheroid formation. Like spheroids, organoid-derived EVs show higher yield and biological activity than those isolated from 2D cultures [107].

*Methods for spheroid formation* These methods can be broadly divided into scaffold-free and scaffold-based approaches. Scaffold-free methods include pellet culture, ultra-low attachment plates, hanging-drop, and magnetic levitation, while scaffold-based methods include hydrogel and solid scaffold encapsulation. Scaffolds can be bioprinted and combined with spinner flasks and bioreactors for large-scale EV production. The specific 3D culture method adopted can significantly affect the yield and functions of EVs. For instance, GelMA microspheres seeded with ADSCs produced seven times more EVs compared to 2D cultures [80]. However, the EV yield from GelMA microspheres was threefold lower than that from spheroids formed by ultra-low attachment culture plates, and their miRNA profiles differed, illustrating how culture conditions influence EV composition and therapeutic potential [80].

Each technique offers unique advantages for supporting stem cell behavior and promoting EV release, which are discussed in detail in the following section.

## Three-dimensional culture techniques: a closer look

### Scaffold-free methods

Scaffold-free methods offer a simple approach to generate 3D cell spheroids, relying on the inherent ability of cells to self-aggregate without the need for an external support matrix. These methods typically involve forcing adherent cells to aggregate through centrifugation, utilizing suspension culture systems such as ultra-low attachment plates (chemically modified with coatings like chitosan or Pluronic F-127, or physically modified with concaved bottoms), or employing the hanging drop technique.

*Pellet culture* In pellet culture, cells are centrifuged to form compact 3D pellets. This method is simple and cost-effective but may cause hypoxia in larger spheroids (> 500 μm in diameter), limiting nutrient diffusion and waste removal. Shear stress from centrifugation can damage cells. It is moderately laborious with low reproducibility, making it unsuitable for large-scale EV production.

*Ultra-low attachment plates* Spheroid formation can also be enhanced by culturing cells in ultra-low-adhesion plates with U-shaped bottom coated with anti-adhesion solution. BMSCs in non-adherent culture can reacquire a more primitive phenotype through metabolic reconfiguration, leading to morphological and functional changes in mitochondria, a shift from oxidative metabolism to glycolysis and the pentose phosphate pathway, and enhanced autophagy [101].

This technique can yield at least 16-fold more EVs from ADSCs compared to 2D cultures [80]. In one study employing methylcellulose hydrogel-coated wells, RNA sequencing revealed that ADSC spheroids formed under inflammatory conditions exhibited significant enrichment of genes associated with immune response, cytokines, and tissue remodeling compared to traditional 2D cells [102]. The mRNA expression of *Igf1*, *Tgfb1*, and *Vegfa* was significantly elevated in the ADSC spheroids compared to cells in 2D culture. Moreover, the secretome of ADSC spheroids demonstrated enhanced promotion of tenocyte proliferation and migration mediated by the ERK signaling pathway, and more effectively inhibited IL-6 and IL-1α secretion by M1 macrophages via the COX-2/PGE2/EP4 signaling pathway, compared to the secretome isolated from 2D culture [102]. In vivo, the injection of ADSC spheroids promoted tendon healing compared to the injection of cells cultured in monolayer in a rabbit Achilles tendon transection and repair model [102]. However, the specific effects of EVs secreted by these spheroids on tendon repair have not yet been reported.

While this method is cost-effective, the non-uniformity of spheroid shape and size, along with moderate labor intensity and low reproducibility, limits its scalability for EV production.

*Hanging drop method* In the hanging drop method, cells aggregate at the tip of a droplet of culture medium, spontaneously forming a spheroid composed of cells embedded in a self- produced matrix. Notably, cell apoptosis during compaction can enhance the production of an anti-inflammatory secretome by human BMSCs [92, 94]. BMSC spheroids formed using this method exhibit higher expression levels of anti-inflammatory molecules, such as tumor necrosis factor-stimulated gene 6 (TSG-6), stanniocalcin-1 (STC-1), and leukemia inhibitory factor (LIF), as well as anti-tumorigenic molecules like interleukin-24 (IL-24) and TNF-related apoptosis-inducing ligand (TRAIL). Additionally, these spheroids show increased expression of BMSC homing receptor C-X-C chemokine receptor type 4 (CXCR4) and lower levels of Wnt signaling inhibitor dickkopf-1 (DKK1) compared to monolayer BMSCs [92].

Furthermore, BMSC spheroids produced via the hanging drop method display fewer cells in the S-phase of the cell cycle and lower surface expression of cell adhesion molecules (CD146 and CD166), along with higher expression of the integrin adhesion subunit CD49b and the metastasis inhibitor CD82 compared to monolayer BMSCs [92].

In a related study, the same group reported that TSG-6 expression increased by over 500-fold, STC-1 by over 10-fold, and COX-2 by over 100-fold, with prostaglandin E2 (PGE2) levels reaching 20–100 ng/mL in BMSC spheroids containing 2.5 × 10^4^ cells/drop [108]. Additionally, the conditioned medium from these BMSC spheroids polarized macrophages from the M1 to the M2 phenotype, shifting their cytokine profile from pro-inflammatory to anti-inflammatory [94, 98]. This anti-inflammatory effect on macrophages was mediated by increased PGE2 levels in the conditioned medium, dependent on caspase and NF-kB activation in BMSCs [94].

Similar to pellet culture, the hanging drop method is simple and does not require specialized or expensive equipment for small-scale applications. However, in both cases, optimizing the pellet or spheroid size for application in target tissues is crucial [109]. Evaporation can dry the droplets and medium exchange can disturb their formation, limiting the duration of cell culture and making large-scale production unfeasible. There are currently no studies on the effects of EVs derived from MSC spheroids formed by the hanging drop method on tendon and TBJ repair. Further research is needed to evaluate the yield and quality of EVs produced by this method for these applications.

*Magnetic levitation* A recent approach involves loading cells with magnetic nanoparticles, such as iron oxide, and applying an external magnetic field to induce aggregation and spheroid formation [110]. This method enables control over cell density and spheroid shape by adjusting the magnetic field. Additionally, the magnetic nanoparticles enable cell tracking and imaging. A previous study has shown that levitated ADSC culture shows enhanced spheroid formation, proliferation and multi-lineage differentiation potential [110]. Further studies to understand the effects of magnetic levitation on the quantity and quality of EVs produced for tendon and TBJ regeneration are warranted.

However, magnetic levitation is laborious and requires specialized equipment, increasing EV production cost. The magnetic nanoparticles may have potential side effects on cellular functions, affecting clinical application. Moreover, low reproducibility makes this method unsuitable for large-scale EV production.

*Challenges and considerations* Scaffold-free methods present technical challenges in establishing and maintaining stable cultures. Spheroid and organoid formation can be variable. Spheroid size can limit oxygen and nutrient diffusion, and affect waste removal. Previous studies have reported that spheroid size and culture duration influence MSC metabolic activity, paracrine potential, and immunomodulatory capacity [92, 111–113]. To prevent cell death in the center of spheroids, careful optimization of size, cell density, and culture duration is crucial. Due to the time-consuming nature of these 3D culture procedures, they are primarily suitable for laboratory-scale spheroid formation. Currently, no studies have employed these methods for stem cell-derived EV production specifically for tendon and TBJ repair.

In summary, scaffold-free methods offer versatile and accessible options for generating cell spheroids. However, careful optimization of culture parameters and further investigation into their application for tendon and TBJ repair are necessary.

### Scaffold-based methods

Scaffold-based 3D culture platforms, constructed from natural or synthetic materials, provide specific functional ligands and a 3D environment that supports cell adhesion and growth. They can be broadly categorized into injectable hydrogels and solid scaffolds, each offering unique advantages for mimicking the native cellular microenvironment.

*Injectable hydrogels* Hydrogels made of cross-linked natural or synthetic polymers can be used as 3D scaffolds for EV production. They are soft and elastic after water absorption, which can be controlled by varying the degree of cross-linking, making them ideal for mimicking the stiffness and mechanical properties of soft tissues. Their porosity and degradation rates can be tailored for controlled release of encapsulated materials. Common natural biomaterials, including collagen, fibrin, Matrigel, alginate, gelatin, agarose, hyaluronic acid, and decellularized ECM, are commonly used to produce injectable hydrogels due to their inherent biocompatibility and ability to promote cell-matrix interactions. Notably, gelatin methacrylate (GelMA), which is gelatin modified with methacryloyl groups, allows precise customization of mechanical strength, degradation rate, porosity, and mesh structure by adjusting photocrosslinking time and gel concentration, providing a versatile platform for EV production.

**Fig. 3:**
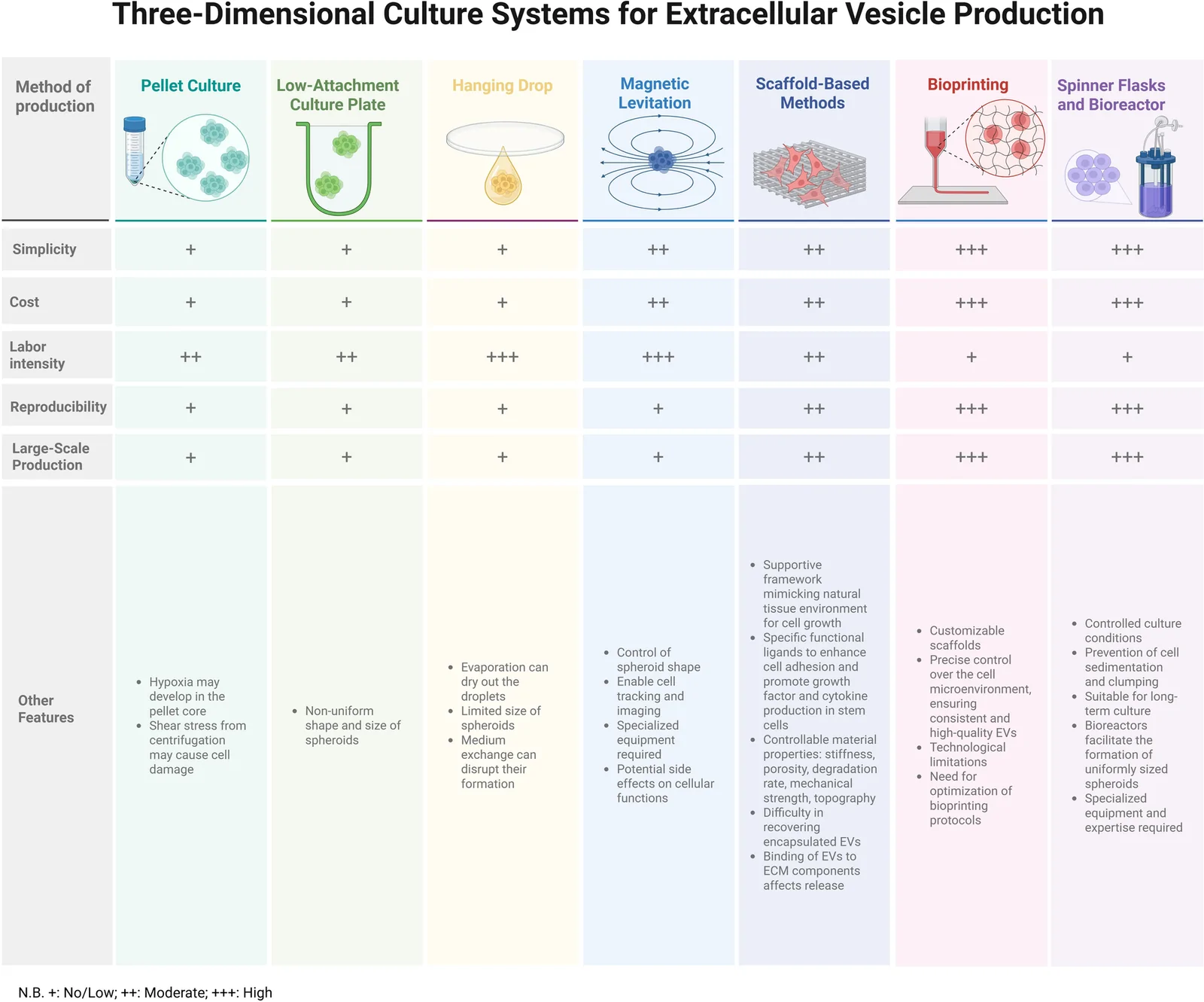
Three-dimensional culture systems for extracellular vesicle production. Created in BioRender. https://BioRender.com/073nt35

**Table 1:** Studies on preconditioned or engineered extracellular vesicles of stem cells on tendon and tendon-to-bone junction repair

Preconditioning or engineering method	Cell type for EV production	In vitro model / effects (compared to untreated group)	In vivo model / effects (compared to untreated group)	Ref.
LIPUS	BMSCs	- BMSCs - Rich in chondrogenic miRNAs (14 out of 21 tested) (e.g., miR-14) - Reduced levels of adipogenic miRNAs (7 out of 11 tested) - Promoted chondrogenic differentiation and inhibited adipogenic differentiation of BMSCs mediated by exosomal miR-140 transfer	- Mouse model of rotator cuff repair - Superior effects in promoting fibrocartilage regeneration and inhibiting fatty infiltration	[39]
Magnetic stimulation	BMSCs	- NIH3T3 fibroblasts - Rich in miR-215p, miR-25-3p, miR-92a-3p, miR-122-5p, miR-125b-5p, and miR-150-5p - Promoted fibroblast proliferation, migration, and fibrogenesis via miR-21-5p-mediated downregulation of SMAD7	- Rat model of ACLR - Superior effects in promoting inhibition of peritunnel bone loss, and increasing bone ingrowth into tendon graft, fibrocartilage formation, mechanical properties of TBJ	[40]
Static magnetic field	ADSCs	- Effects on target cells not examined - Increased production of microvesicles rich in VEGF, BMP-2, p53 and reduced level of TNF-α	–	[41]
IFN-γ and TNF-α pretreatment	BMSCs	- Activated rat splenocytes - Differential inflammation-related cytokine profiles rich in ICAM1, CXCL12, CCL5 and expressed low levels of IL-5, IL-6, IL-10, and IL-13 - Rich in long noncoding RNA sequences and 11 miRNAs (> 10-fold change)-related to immunomodulation - Rich in Cox-2 that enhanced inhibition of TNF-α and IFN-γ release from activated splenocytes	–	[47]
IFN-γ pretreatment	ADSCs	- Macrophages and tendon cells - Rich in miR-147b - Superior effects in inhibiting inflammatory response in macrophages via miR-147b-mediated inhibition of TLR4/NF-kB signaling pathway - Superior effects in enhancing tendon cell proliferation and collagen production	- Mouse model of Achilles tendon 2/3 transection and repair - Superior effects in attenuating early tendon inflammatory response, reducing tendon gap-rupture rate, enhancing collagen formation, and hence promoting repair	[18, 49]
Melatonin pretreatment	ADSCs	- Macrophage, and BMSCs, human rotator cuff tear tissue - Increased yield and anti-inflammatory capacity - Rich in platelet factor 4 protein - Superior effects in inhibiting M1 macrophage polarization through platelet factor 4 transfer-mediated activation of cAMP signaling pathway - Superior effects in promoting M2 macrophage polarization and reducing inflammation in fresh human rotator cuff tear samples - Superior effects of alleviating suppressive effect of M1 macrophages on migration and chondrogenic differentiation of BMSCs	- Rat model of rotator cuff repair - Superior effects in mitigating inflammation, promoting fibrocartilage regeneration and hence facilitating TBJ repair	[50]
Kartogenin pretreatment	BMSCs	-	- Rat model of chronic rotator cuff repair - Superior effects in promoting fibrocartilage regeneration, collagen maturation, mechanical properties and hence TBJ healing	[51]
Eugenol pretreatment	BMSCs	- TDSCs - Superior effects in increasing viability, PCNA expression, colony number, and reducing apoptosis, ROS, and anti-oxidant levels of H_2_O_2_-treated TDSCs - Protected TDSCs against H_2_O_2_-induced oxidative stress via Nrf2/HO-1 pathway	- Rat model of patellar tendon injury - Superior effects of *pre-treated TDSCs* in promoting tenogenesis and matrix deposition, and hence tendon healing	[53]
Hydroxycamptothecin pretreatment	HUMSCs	- Fibroblast cell line - Rich in ERS-associated proteins (GRP78, CHOP), pro-apoptotic proteins (Bax) and expressed reduced levels of anti-apoptotic proteins (Bcl2) - Superior effects in inhibiting proliferation, viability, and myofibroblast differentiation of TGF-β1-treated fibroblasts mediated by activation of ERS pathway	- Rat model of partial Achilles tendon transection and repair - Superior effects in improving tendon healing, and suppressing myoblast activation - Insignificant improvement in tendon adhesion and no difference in mechanical properties	[37]
Bioactive glass pretreatment	ADSCs	- Macrophages - Expressed distinct miRNA profile with enhanced anti-inflammatory and regenerative effects - Rich in miR199b-3p and miR-125-5p that may contribute to the superior effects in M2-macrophage-mediated angiogenesis	- Rat model of full-thickness Achilles tendon rupture and repair - Superior angiogenic effects, likely attributed to increased M2 macrophage polarization - Superior tenogenic effects and biomechanical properties, and hence promoted tendon repair	[57]
miR-337-3p overexpression	TDSCs	- Tenocytes - Superior effects in promoting proliferation and migration of tenocytes - Superior effects in downregulating caspase 3 and protecting tenocytes from apoptosis	–	[58]
miR-29a-3p overexpression	HUMSCs	- TDSCs - Superior effects in promoting expression of tenogenic markers in TDSCs via downregulation of PTEN, activation of Akt, and consequently upregulating mTOR signaling and TGF-β1 production	- Rat model of Achilles tendon transection and repair - Superior effects in promoting tenogenesis but displayed no further improvement in biomechanical properties	[31]
miR-21a-3p antagonist overexpression	HUMSCs	- Fibroblast cell line - Superior effects in reducing the expression levels of p-p65, Cox-2, Col III, α-SMA in TGF-β1-treated fibroblasts	–	[13]
Scx overexpression	PDGFRα(+) BMSCs	- Pre-osteoclasts - Reduced EV numbers - Enrichment of miRNAs involved in osteoclast differentiation pathway - Rich in miR-6924-5p - Superior effects in inhibiting osteoclastogenesis of pre-osteoclasts via miR-6924-5p-mediated inhibition of CXCL12 and OCSTAMP expression	- Mouse extra-articular tendon-bone tunnel model - Superior effects in reducing osteoclastogenesis at peritunnel bone and enhancing biomechanical properties of TBJ via miR-6924-5p delivery	[34]
CD63-CBD overexpression	TDSCs	- TDSCs and BMSCs - Superior effects in enhancing proliferation and tenogenic differentiation of TDSCs and BMSCs	- Rat model of Achilles tendon segmental defect and repair - Superior effects in promoting endogenous stem cell recruitment, M2 macrophage polarization, tenogenesis, histological score, collagen deposition, collagen fibril diameters, biomechanical properties, and Achilles tendon functional index - Reduced expression of inflammatory cytokines	[67]
LDN193189-pretreatment and CBD-modification	CD26^+^ TDSCs	- BMSCs - Reduced osteogenic marker *Runx2* and has no effect on the tenogenic marker *Scx* - Both LDN193189-pretreated and CBD-modified EVs, as well as LDN193189-pretreated EVs 3D-bioprinted in collagen type 1/PLGA scaffolds, promoted similar expression of *Scx* in BMSCs and has no effect on *Runx2* expression	- Aged rat model of segmental Achilles tendon defect - Superior effects in promoting tendon repair and preventing heterotopic ossification - More effective than scaffolds with only LDN193189-pretreated EVs, which outperformed untreated EVs and the injury-only group	[69]
Kartogenin direct loading	BMSCs	- BMSCs - Increased expression of osteogenic, chondrogenic and tenogenic markers in BMSCs - Enhanced *Mospd* expression and chondrogenic marker expression in BMSCs	- Rat model of rotator cuff repair - Promoted fibrocartilage regeneration, collagen fiber arrangement, and biomechanical strength	[70]
Hybrid nanocarriers prepared by sonication for collagen type I mRNA delivery	ADSCs	- TDSCs from pathological human Achilles tendon - Increased production of PICP in TDSCs	–	[71]
Hypoxia	ADSCs from subcutaneous fat around shoulder joint	- Effects on target cells not examined - Rich in proteins associated with ECM homeostasis including MMP2, COL6A, CTSD, TNC	–	[75]
Hypoxia	BMSCs	- HUVECs - Superior effects in promoting proliferation, migration, and tube formation of HUVECs	- Rat model of ACLR - Superior effects in tunnel bone formation, angiogenesis and histological score of TBJ healing, and biomechanical properties	[27]
Dynamic perfusion bioreactors	TDSCs cultured in GDF-5-supplemented medium	- Effects on target cells not examined - 2.6-fold higher total protein in EVs cultured under dynamic condition - CD81 and TSG-101 were detectable only in dynamic culture - Higher surface expression of CD63, CD1c, CD105, CD41b, CD49e, CD62P, MCSP, CD2, CD3 and CD56 in dynamic culture - Equal surface expression of HLA-DR/DP/DQ, CD25 in dynamic culture compared to static culture - Surface expression of SSEA-4, CD9 was detectable only in static culture while surface expression of CD45 was detected only in dynamic culture	–	[116]

*N.B. LIPUS* Low-Intensity Pulsed Ultrasound, *BMSCs* Bone Marrow Stromal Cells, *SMAD7* SMAD Family Member 7, *ACLR* Anterior Cruciate Ligament Reconstruction, *TBJ* Tendon-to-Bone Junction, *ADSCs* Adipose-Derived Stromal Cells, *VEGF* Vascular Endothelial Growth Factor, *BMP-2* Bone Morphogenetic Protein 2, *TNF-α* Tumor Necrosis Factor Alpha, *ICAM1* Intercellular Adhesion Molecule 1, *CXCL12* C-X-C Motif Chemokine Ligand 12, *CCL5* C-C Motif Chemokine Ligand 5, *IL-5* Interleukin 5, *IL-6* Interleukin 6, *IL-10* Interleukin 10, *IL-13* Interleukin 13, *Cox-2* Cyclooxygenase-2, *TLR4/NF-kB* Toll-Like Receptor 4/Nuclear Factor kappa-light-chain-enhancer of activated B cells, *cAMP* Cyclic Adenosine Monophosphate, *TDSCs* Tendon-Derived Stem / Progenitor Cells, *PCNA* Proliferating Cell Nuclear Antigen, *ROS* Reactive Oxygen Species, *H*_*2*_*O*_*2*_ Hydrogen Peroxide, *Nrf2/HO-1* Nuclear Factor Erythroid 2-Related Factor 2/Heme Oxygenase 1, *HUMSCs* Human Umbilical Mesenchymal Stem Cells, *ERS* Endoplasmic Reticulum Stress, *GRP78* Glucose-Regulated Protein 78, *CHOP* C/EBP Homologous Protein, *Bax* Bcl-2-Associated X Protein, *Bcl2* B-Cell Lymphoma 2, *TGF-β1* Transforming Growth Factor Beta 1, *PTEN* Phosphatase and Tensin Homolog, *Akt* Protein Kinase B, *mTOR* Mechanistic Target of Rapamycin, *Col III* Collagen Type III, *α-SMA* Alpha-Smooth Muscle Actin, *Scx* Scleraxis, *PDGFR*α Platelet-Derived Growth Factor Receptor Alpha, *CXCL12* C-X-C Motif Chemokine Ligand 12, *OCSTAMP* Osteoclast-Stimulating Factor, *CBD* Collagen Binding Domain, *Runx2* Runt-Related Transcription Factor 2, *PLGA* Poly(lactic-co-glycolic acid), *PICP* Procollagen Type I C-Terminal Propeptide, *ECM* Extracellular Matrix, *MMP2* Matrix Metalloproteinase 2, *COL6A* Collagen Type VI Alpha, *CTSD* Cathepsin D, *TNC* Tenascin C, *HUVECs* Human Umbilical Vein Endothelial Cells, *GDF-5* Growth Differentiation Factor 5, *MCSP* Melanoma Chondroitin Sulfate Proteoglycan, *HLA-DR/DP/DQ* Human Leukocyte Antigen – DR/DP/DQ, *SSEA-4* Stage-Specific Embryonic Antigen 4

All relevant papers in Pubmed were searched and last update was done on 18 May 2025

Compared to scaffold-free methods, natural hydrogels provide a supportive framework that closely mimics the natural ECM. The ECM-based components in natural hydrogels enhance growth factor and cytokine production in MSCs compared to 2D cultures [114]. For example, HUMSCs embedded in sodium alginate and cultured in a 3D environment showed a remarkable 3-fold increase in EV production [115]. These EVs exhibited elevated levels of VEGF, promoted the migration and proliferation of target MSCs and inflammatory chondrocytes, and suppressed cartilage-degrading factors more effectively than EVs from 2D cultures [115]. Furthermore, BMSCs encapsulated in sodium alginate / hyaluronic acid hydrogel produced approximately 3.9 times more EVs than conventional 2D culture [116]. Additionally, the EV yield of human periodontal ligament stem cells cultured in collagen gel in a 3D environment was 2.5-fold higher compared to cells cultured in the traditional 2D monolayer [117]. These 3D-derived EVs significantly promoted proliferation, migration, and osteogenic marker expression of target BMSCs compared to their 2D counterparts [117]. In addition, synovial fluid-derived cells seeded in gelatin crosslinked with transglutaminase in 3D culture showed higher viability and lower senescence compared to cells cultured in monolayer. Their EVs also exhibited superior effects on chondrogenic differentiation of human chondrocytes compared to EVs derived from 2D cultures [95]. The effects of EVs produced by stem cells encapsulated in natural hydrogels on tendon and TBJ repair remain unexplored.

*Solid scaffolds* Solid scaffolds provide a well-defined, physiologically relevant 3D environment for EV production. Notably, the yield of EVs derived from 3D cultures of HUMSCs in chitin-based polysaccharide fibers was 1.6-fold and 1.17-fold higher than those from 2D cultures and 3D cultures in U-bottom plates, respectively [96]. The EVs from these chitin-based cultures exhibited a distinct protein profile compared to those from 2D cultures. Further transcriptomic investigations revealed that 3D cultures enhanced the expression of genes associated with stemness, migration, and EV production. This enhancement was validated by the upregulation of key stemness genes such as Octamer-binding transcription factor 4 (*OCT4*), Nanog homeobox (*NANOG*), and stage-specific embryonic antigen 4 (*SSEA4*), along with improved migration capacity in 3D-cultured HUMSCs [96].

Micrcoarriers are beads made from various materials, sizes, and surface topographies that provide a substrate for cell adhesion in bioreactors, which can be kept in suspension through agitation. Cells can grow on the surface or inside microcarriers depending on pore size. The stiffness, hydrophobicity, permeability, and topography of microcarriers affect the biological activities of the seeded cells and their EV production. Bulk RNA sequencing revealed higher expression of tenocyte markers in TDSCs cultured in 3D gelatin microcarriers compared to those in 2D culture [118]. The use of microcarriers enhanced tenogenesis, and their transplantation facilitated tendon healing via activation of the integrin-FGF7-ERK/MAPK/PI3K/STAT signaling pathway [118]. However, the effects of EVs derived from this 3D culture were not examined in this study, highlighting an area for further investigation.

The behavior and secretory profile of MSCs can be influenced by the surface topography, porosity, and mechanical strength of the biomaterials used in scaffold construction. The arrangement and stiffness of microfibers, for example, affect the production of paracrine factors. Conditioned medium from BMSCs cultured on aligned, random, or mesh-like fibrous electrospun scaffolds produced different paracrine factors, with conditioned medium produced from mesh-like structures showing higher anti-inflammatory effects [119]. Additionally, MSCs cultured on Ti-alloy with scale-textured surfaces showed minimal pro-inflammatory cytokine production compared to MSCs cultured on a smooth surface [120]. Moreover, matrix elasticity also affected MSC fate [121].

While natural biomaterials are generally preferred for EV production due to their high cell affinity and low cytotoxicity, they suffer from low stiffness and poor mechanical strength that affect the properties of EVs. Synthetic polymers offer stiffer materials, providing higher mechanical strength and the ability to customize porosity. However, they are often limited by lower biocompatibility and a lack of cell adhesive sites. To overcome these limitations, researchers are developing hybrid scaffolds that combine both synthetic and natural materials for the production of therapeutic EVs.

*Challenges and considerations* A notable challenge of many scaffold-based 3D culture platforms, particularly hydrogels, is their gel-like structure, which can be difficult to dissolve, complicating efficient recovery of encapsulated EVs and cells for downstream analysis. Other challenges include the difficulty of isolating EVs from a 3D scaffold with smaller pores and the potential binding of EVs to ECM components, which can hinder EV release. Further processing, such as enzymatic digestion of scaffolds, may be needed to retrieve EVs, but this can potentially affect their bioactivity. The scaffold materials add to the cost of EV production. Despite the moderate reproducibility of scaffold-based methods, they are still not fully adaptable for large-scale EV production.

In summary, scaffold-based 3D culture platforms present promising avenues for optimizing the EV production environment. Careful consideration of material properties, culture conditions, and downstream processing techniques is crucial for maximizing EV yield, purity, and bioactivity. While promising data exists, data on the specific effects of EVs generated by 3D culture of stem cells in scaffolds on tendon and TBJ repair are lacking, representing a significant gap in knowledge that needs to be addressed. Moreover, further research is needed to fully elucidate the complex interplay between cells, scaffolds, and EVs to optimize these techniques for clinical translation.

### 3D bioprinting

3D bioprinting facilitates the creation of scaffolds with varying nano-topologies that guide stem cell organization, mimicking native tissues and enabling scalable EV production.

In this process, scaffolds can be printed for cell culture, or a “bio-link” can be formed by combining cells, growth factors, and structural biomaterials to create cell constructs. This precise control over construct size, cell density, and positioning within the culture dish enhances the consistency and quality of EV produced.

For example, coaxial 3D printing of meter-long hollow hydrogel microfibers encapsulating BMSCs resulted in a 1009-fold increase in EV production while preserving pro-angiogenic properties compared to traditional 2D culture [122]. Additionally, EVs derived from BMSCs cultured on 3D-printed hydroxyapatite scaffolds demonstrated superior effects on cell proliferation, migration, tube formation, and angiogenesis in vivo, outperforming EVs isolated from 2D cultures [123].

Furthermore, Yan et al. showed that EVs isolated from HUMSCs cultured on 3D-printed acellular cartilage ECM scaffolds exhibited enhanced abilities to regulate stem cell behavior, chondrogenesis, and immunomodulation, as well as osteochondral defect repair, compared to EVs isolated from cells cultured on 2D acellular ECM films [106]. Despite these promising results, the application of 3D bioprinting for generating EVs specifically for tendon and TBJ repair remains largely unexplored and requires further investigation.

*Challenges and considerations* Key challenges include high costs and technological limitations in the bioprinting process. The bioprinting protocols for different cell types and scaffold materials to enhance EV yield and functionality need to be optimized.

In summary, 3D bioprinting offers significant advantages for enhancing EV production. While current studies highlight its potential, further research is essential to investigate its applications in tendon and TBJ repair.

### Spinner flasks and bioreactors

Spinner flasks and bioreactors enable scalable 3D cell culture systems for efficient EV production in clinical applications.

*Spinner flasks* In spinner culture systems, cell suspensions are gently and continuously agitated using a mixer. It enhances uniform exposure of cultured cells to oxygen and nutrients, alleviates challenges such as cell sedimentation and clumping, and promotes a favorable environment for long-term cell growth and larger scale production. Notably, dynamic 3D culture of MSC spheroids in spinner flasks improves osteogenic and adipogenic differentiation potential compared to monolayer culture [93]. However, spheroid size can be non-uniform, and shear stress from spinning may damage the cultured cells.

*Bioreactors* Perfusion bioreactors offer continuous media circulation, enhancing scalability in EV manufacturing. This setup facilitates nutrient and oxygen transport and waste removal, leading to increased cell viability and metabolic activity. Scaffold-free methods and microcarriers are commonly used in bioreactors to produce more uniform cell aggregates. Importantly, the shear stress generated by fluid flow modulates the regenerative properties of MSCs and their EVs [124–126].

ADSC spheroids encapsulated in collagen gel and cultured in bioreactors exhibited superior osteogenic differentiation potential under osteogenic induction compared to traditional 2D culture [127]. Compared to static culture, BMSCs cultured in perfusion bioreactors demonstrated significant increases in EV protein content—5.7-fold at a flow rate of 0.1 mL/min and 7-fold at 1 mL/min [124].

A dynamic hollow fiber bioreactor consists of numerous small, semi-permeable hollow fibers bundled together within a tubular shell. Cells are seeded on the outer surface of the hollow fibers, while complete culture medium circulates through the lumen. At the same time, serum-free medium flows through the extracellular space between the fibers, allowing EV accumulation. The fibers protect cells from shear stress, and separate flow of media for nourishing the cells and facilitating EV secretion that help prevent cross-contamination with other proteins. HUMSC cultured in a hollow fiber bioreactor produced 5.7 times more EVs than in 2D culture [128]. The EVs isolated from the 3D system showed stronger stimulatory effects on chondrocyte proliferation, migration, and matrix synthesis, as well as apoptosis inhibition. They also showed superior therapeutic effects on osteochondral repair compared to 2D cultures [128]. Furthermore, dynamic hollow fiber bioreactor systems have shown to increase BMSC-EV production while maintaining stem cell function under a current Good Manufacturing Practice (cGMP)-compliant procedure, indicating their potential for clinical scalability [129].

In tendon and TBJ research, only one study has examined the effects of dynamic perfusion on EV production from human TDSCs cultured in growth-differentiation factor-5 (GDF-5)-supplemented medium [130]. This approach yielded approximately 2.6 times more total protein from EVs compared to static cultures in the same tenogenic differentiation medium [130]. No in vivo experiments were conducted. Further research is necessary to assess the therapeutic effects of these EVs on tendon and TBJ regeneration.

Regarding the molecular mechanisms, fluid flow from bioreactors increases intracellular calcium and calcium-related proteins, which may explain the increase in EV biogenesis [124].

*Challenges and considerations* The complexity of spinner flasks and bioreactors requires careful monitoring and maintenance, which can impose significant costs. Moreover, specialized knowledge is necessary for their operation, limiting access for some research facilities.

In summary, despite the challenges associated with spinner flasks and bioreactors, their ability to provide scalable EV yields and improve cell viability positions them as essential tools for future EV research and clinical applications.

An overview of various 3D systems for EV production, along with their advantages and limitations, is shown in Fig. 3.

## Limitations of current studies and future research directions

EV yield poses a significant limitation to the clinical translation of therapies for tendon and TBJ repair. Various strategies have been employed to increase EV production, enhance their targeting capabilities, extend circulation time, and improve drug loading, thereby enhancing therapeutic effects.

Our systematic literature search, although limited to a single database that covers over 70% of scientific biomedical publications, indicates that research on EV enhancement strategies for tendon and TBJ healing is still in its infancy. Most studies, except those on unmodified EVs, have focused on acute tendon and TBJ tear and repair models, underscoring the need for further investigation into the effects of functionally modified EVs in degenerative tendon repair.

While some studies have examined the effects and molecular mechanisms of EVs in promoting tendon and TBJ repair, the impact of functional modifications—particularly those derived from stem cells cultured in modified environments or subjected to surface or content alterations—remains largely unexplored. Most reported studies have not profiled the cargo content of functionally modified EVs, which is essential for understanding the mechanisms underlying improved yield and functionality. Identifying active components in EVs is crucial for quality control in clinical applications, optimizing outcomes of EV-based therapies, and developing innovative strategies for accelerating tendon and TBJ healing. Moreover, multi-omics analyses of tendons and TBJ tissue following EV treatment are needed to clarify molecular mechanisms, particularly in reducing tendon fibrosis and promoting fibrocartilage regeneration.

The following up times in animal models have been short. Potential immunogenic responses and safety profiles of EVs have not been thoroughly evaluated. Future studies should focus on the long-term benefits and safety of EV therapy for tendon and TBJ healing. Monitoring the biodistribution of EVs after delivery is essential for understanding their behavior in the body.

Importantly, there have been no clinical studies on the effects of EVs isolated from stem cells for treating tendinopathy [131], which should be prioritized in future research. Establishing protocols for standardization, automation, quality control, and validation for large-scale, cost-effective EV production is necessary. The EV treatment protocol—including cell source, dose, administration time, and biomaterials for delivery—must be optimized for clinical translation. Finally, exploring the effects of various combination therapies on enhancing therapeutic EV production and function warrants further investigation.

## Data Availability

All the data included in the manuscript are available on request.

## Abbreviations

EV: Extracellular vesicles
TBJ: Tendon-to-bone junction
3D: Three-dimensional
MSC: Mesenchymal stromal cell
TDSC: teNdon-derived stem/progenitor cell
iPSC: Induced pluripotent stem cell
ADSC: Adipose-derived stromal cell
HUMSC: Human umbilical cord MSCs
ECM: Extracellular matrix
PTEN: Phosphatase and tensin homolog
Akt: Protein kinase B
mTOR: Mammalian Target of Rapamycin
TGF-β1: Transforming growth factor-beta 1
Cox-2: Cyclooxygenase-2
Col3A1: Collagen 3 A 1
α-SMA: Alpha-smooth muscle actin
RhoA: Ras homolog family member A
Scx: Scleraxis
PDGFRα: Platelet-derived growth factor receptor alpha
MMP2: Matrix metalloproteinase 2
GRP7: Glucose-regulated protein 7
CHOP: C/EBP homologous protein
Bax: Bcl-2 associated X protein
BMSC: Bone marrow-derived stromal cell
MMP14: Matrix metalloproteinase 14
LIPUS: low-intensity pulsed ultrasound
VEGF: Vascular endothelial growth factor
BMP-2: Bone morphogenetic protein-2
TNF-α: Tumor necrosis factor-alpha
TLR: Toll-like receptor
IFN-γ: Interferon-gamma
Cox-2: Cyclooxygenase-2
HGF: Human growth factor
IDO: Indoleamine 2,3-dioxygenase
PCL/SF: Electrospun polycaprolactone/silk fibroin
IL-1β: Interleukin-1beta
TLR4/NF-kB: Toll-Like Receptor 4/Nuclear Factor kappa-light-chain-enhancer of activated B cells
cAMP: Cyclic adenosine monophosphate
LPS: Lipopolysaccharide
Nef: Negative regulatory factor
LAMP2B: Lysosomal associated membrane protein 2B
CBD: Collagen-binding domain
Mospd: Mosaic protein domain
PEG: Polyethylene glycol
PICP: Procollagen type I carboxy-terminal peptide
Col6A: Collagen type 6 A
CTSD: Cathepsin D
TNC: Tenascin C
2D: Two-dimensional
TSG-6: Tumor necrosis factor-stimulated gene 6
STC-1: Stanniocalcin-1
LIF: Leukemia inhibitory factor
IL-24: Interleukin-24
TRAIL: TNF-related apoptosis-inducing ligand
CXCR4: C-X-C chemokine receptor type 4
DKK-1: Dickkopf-1
PGE2: Prostaglandin E2
GelMA: Gelatin methacrylate
Oct4: Octamer-binding transcription factor 4
Nanog: Nanog homeobox
SSEA4: Stage-specific embryonic antigen 4
IL-24: Interleukin-24
cGMP: Current Good Manufacturing Practice
GDF-5: Growth-differentiation factor-5
YAP: Yes-associated protein
NSAID: Non-steroidal anti-inflammatory drugs
